# Sister Children's

**Published:** 1918-02-02

**Authors:** 


### SOME HOSPITAL TYPES OF BRITISH NURSING.

### V.—Sister Children's.

PICTURE a slight woman of medium height, grey hair, the kindest pair of grey eyes imaginable, and a clear complexion in spite of fifty-one years. A stranger might have passed her by in the street as a commonplace woman, but what a wealth of gold was in her heart!

It was my good fortune to work with her as junior sister some years ago, and the effect produced on my mind has never been effaced. The children's ward in which she had toiled for twenty-five years contained thirty-six cots, but often had to accommodate from four to ten emergencies in addition, made as comfortable as possible in spinal carriages, couches, or even on two arm-chairs placed together. I think of her first as woman and next as nurse. From the first point of view it was noticeable that none of us came into contact with her without feeling the better for it. Always a better tone was observable in the sisters' sitting-room if she were present. By her ready advice and broad-minded common sense all the many worries and difficulties I experienced in my work were smoothed away like magic. If any of us were in doubt it was always to her we confided our cares, and in our worst troubles her kindly pat on the shoulder made us feel that life was not so bad after all. She could tell a story well, and awake refreshing peals of laughter over experiences of past days.

But even when off duty her mind was never far from the children. Round them centred the real interest of her life, and her busy fingers were always occupied with some bed-jacket or other comfort for her charges.

As a sister she was a firm disciplinarian, both with her little patients and with her probationers. Her ward was the brightest and best-cared-for in the hospital. No slackness was tolerated, and it was a red-letter day in a nurse's training to receive a good report from Sister after her spell of work in the children's ward. Many a nurse who had failed under other sisters was sent to this ward with the knowledge on Matron's part that Sister would discover any latent abilities she might possess. Owing to her reputation for strictness, probationers went to her ward in fear and trembling, but nearly all of them came to admire and love her, and left her full of gratitude for the training they had received. Although her knowledge of children's nursing was exceptional she was always willing to learn, but she could be very sarcastic with the younger residents on occasion, and these incidents generally ended by the young surgeon or doctor coming round to her opinion and sitting at her feet. Even members of the honorary medical staff would refer knotty points to her, and get (so they said) many a wrinkle from her long experience.

Hers had not been a life of luxury. She once confided to me that she had never been in receipt of more than £35 a year, and out of this had helped to support an invalid mother, had belonged to the pension fund, and taken an occasional modest holiday. This with a smile and no thought that life might have been brighter for her had her pay been higher.

Such was the life of a very humble-minded woman, whose work never took her into the limelight, and was noticed only by the few with whom she came in contact. Yet it is impossible to estimate the extent to which her influence radiated for good among her probationers, among her beloved little patients, and among the mothers in the town, some of whom had been her patients themselves and eagerly sought her advice.

I remember one Christmas Eve helping her to finish the Christmas Tree, and as she moved about with a hare-lip baby newly operated on in her arms she told me she had decorated the tree for twenty years. Truly a fine record in a restless age.

☞ Previous articles appeared on Oct. 27, Dec. 1, Dec. 22, and Jan. 19.

